# A variant of green fluorescent protein exclusively deposited to active intracellular inclusion bodies

**DOI:** 10.1186/1475-2859-13-68

**Published:** 2014-05-16

**Authors:** Govindan Raghunathan, Ganapathiraman Munussami, Hyojin Moon, Hyun-jong Paik, Seong Soo A An, Yong-Sung Kim, Sebyung Kang, Sun-Gu Lee

**Affiliations:** 1 Department of Chemical Engineering, Pusan National University, Busan, South Korea; 2 Department of Polymer Science and Engineering, Pusan National University, Busan, South Korea; 3 Department of Bionanotechnology, Gachon University, Seongnam, South Korea; 4 Department of Molecular Science and Technology, Ajou University, Suwon, South Korea; 5 Department of Biological Sciences, School of Life Sciences, Ulsan National Institute of Science and Technology (UNIST), Ulsan, South Korea

**Keywords:** Green fluorescent protein, Active inclusion bodies, Protein aggregation, Fluorescent particle, Protein folding

## Abstract

**Background:**

Inclusion bodies (IBs) were generally considered to be inactive protein deposits and did not hold any attractive values in biotechnological applications. Recently, some IBs of recombinant proteins were confirmed to show their functional properties such as enzyme activities, fluorescence, etc. Such biologically active IBs are not commonly formed, but they have great potentials in the fields of biocatalysis, material science and nanotechnology.

**Results:**

In this study, we characterized the IBs of DL4, a deletion variant of green fluorescent protein which forms active intracellular aggregates. The DL4 proteins expressed in *Escherichia coli* were exclusively deposited to IBs, and the IBs were estimated to be mostly composed of active proteins. The spectral properties and quantum yield of the DL4 variant in the active IBs were almost same with those of its native protein. Refolding and stability studies revealed that the deletion mutation in DL4 didn’t affect the folding efficiency of the protein, but destabilized its structure. Analyses specific for amyloid-like structures informed that the inner architecture of DL4 IBs might be amorphous rather than well-organized. The diameter of fluorescent DL4 IBs could be decreased up to 100–200 nm by reducing the expression time of the protein *in vivo*.

**Conclusions:**

To our knowledge, DL4 is the first GFP variant that folds correctly but aggregates exclusively *in vivo* without any self-aggregating/assembling tags. The fluorescent DL4 IBs have potentials to be used as fluorescent biomaterials. This study also suggests that biologically active IBs can be achieved through engineering a target protein itself.

## Background

Production of heterologous proteins in bacterial cells often results in the formation of inclusion bodies (IBs), pseudo-spherical particles with size ranging from nanometer to micrometer in diameter [1]. In general, IBs are misfolded and inactive protein deposits, which have been considered to be the waste by-product of protein expression [2,3]. A number of studies were carried out to prohibit the formation of IBs in the process of target protein production. For instance, control of target protein expression rate, engineering of target protein, and co-expression of molecular chaperones were effective in the suppression of IBs formation [4,5].

Recently, the understanding of IBs has been completely changed. IBs of some recombinant proteins were demonstrated to be active, and their biotechnological applications were suggested. For instance, the IBs of enzymes such as amidase, acylphosphatase, and D-amino acid oxidase showed catalytic activities [6-9]. The active enzyme IBs are promising in industrial biotransformation because they can be easily recovered and reused. Other examples include the functional IBs for diagnosis, tissue engineering and for nano-medicines [10-12]. These examples evidently indicate the importance and potentials of active IBs for research and industrial applications.

Green fluorescent protein (GFP) belongs to a family of fluorescent proteins, functionally active if the β-barrel structure composed of 11 β-stands and a single central helix is properly formed [13]. The folding efficiency of wild-type GFP was very low, and hence its production generally led to the formation of IBs mostly composed of misfolded proteins [14]. The engineering of its sequence permitted the generation of various GFP mutants which fold more efficiently to be its active form [15]. Indeed, such GFP variants with high folding efficiency have been widely employed in various scientific and technological fields. On the other hand, recent studies demonstrated that it was possible to achieve biologically active fluorescent IBs of GFP. General GFP IBs exhibited fluorescence because properly folded active proteins were included in the aggregation process of misfolded protein molecules [14]. The formation of active GFP IBs could be further induced by expressing the GFP fused to self-aggregating/assembling peptide or protein sequences [16-19]. It was proposed that such active IBs of GFP could be a good precursor for the preparation of fluorescent biomaterials [20,21].

In our recent study, various GFP deletion mutants were generated based on the structurally stabilized GFP [22]. In the process of screening functional deletion mutants, we serendipitously discovered that DL4, a mutant devoid of an internal loop region, showed high fluorescent activity in the insoluble fraction of cell lysates. This made us to speculate that the deletion may induce the intracellular aggregation of folded DL4 leading to formation of active GFP IBs. Here, the hypothesis was demonstrated by executing various analytical studies. Expression study and confocal microscope analysis confirmed that DL4 was exclusively deposited to IBs in *E. coli* cytoplasm and the IBs were mostly composed of active proteins. We also investigated various biophysical properties of the DL4 active IBs such as spectral characteristics, refolding kinetics, thermal stability, and amyloid-like structural property. Finally, an attempt to prepare fluorescent protein nanoparticles was made by controlling the size of fluorescent DL4 IBs *in vivo*.

## Methods

### Expression and purification of proteins and IBs

The genes encoding DL4 or GFP-hs1 cloned in pET30b(+) were expressed in *E .coli* BL21 (DE3) as previously [22]. The soluble protein fraction of GFP-hs1 was purified by Ni-NTA column chromatography (GE Healthcare Bio-Sciences, Sweden) by using the standard protocol. The IBs of DL4 were purified as per the modified protocol based on previous procedures [23,24]. Cells were lysed by osmotic lysis with Tris-sucrose buffer (50 mM Tris, 735 mM sucrose, 1 mM EDTA, 0.1% sodium azide, 10 mM DTT, pH 8.0) and the lysate was clarified by centrifuging at 6200 g for 10 min. The insoluble pellet was resuspended in Tris buffer (50 mM Tris, 200 mM sodium chloride, pH 8.0) containing DNase (10 μg/ml), lysozyme (0.2 mg/ml), 1 mM PMSF (phenylmethanesulfonylfluoride) and incubated at 37°C for 30 min. The lysate was centrifuged at 15000 g for 10 min and the pellet was resuspended in washing buffer (50 mM Tris, 50 mM sodium chloride, 1% Triton X-100, 1 M urea, 1 mM EDTA, pH 8.0). The suspension was centrifuged at 15000 g for 10 min and the detergent was removed by washing with sterile distilled water and then with the Tris buffer. Finally the purified IBs were stored at -20°C for later use.

### SDS-PAGE analysis

The overexpressed cells were harvested with cell density of 3.0 O.D_600nm_ by centrifuging at 1200 g for 10 min at 4°C. The cell pellets were resuspended in 50 mM Tris buffer (pH8.0), and lysed by French press. The soluble fraction clarified from insoluble fractions was separated by centrifuging the cell lysate at 3700 g for 30 min, and the soluble and insoluble fractions were analyzed by 12% SDS-PAGE using standard protocol.

### Analyses of fluorescent spectral properties, refolding kinetics and stability

The fluorescence was recorded using Perkin Elmer/Wallac Victor 2 Multilabel Counter (1420–011) with excitation and emission at 485 nm and at 515 nm respectively. To determine the specific fluorescence activities of the GFP-hs1 and DL4, the protein concentrations of purified GFP-hs1 and DL4 in the purified IBs were estimated by Bradford’s method and known amounts of the samples were used for the measurement of fluorescence. The ex/em spectra were scanned using Hitachi fluorescence spectrophotometer F-7000 as previously reported [22]. The relative quantum yield of GFP-hs1 and DL4 were estimated by comparing their fluorescence with fluorescein (Sigma) as reference standard in 0.1 M Tris (pH 8.0). The samples and standard fluorescein (0.92) were diluted in 0.1 M Tris buffer to prepare solution of equal absorbance. The emission spectra were recorded by exciting at 490 nm, from 450 nm to 650 nm at a scan speed of 240 nm/min with ex/em slit of 5 nm. The integrated fluorescence intensity was calculated from the emission spectra and the relative quantum yield was calculated as previously described [25]. The refolding kinetics of the GFP-hs1 was measured as reported previously [22]. For refolding plot of DL4, the purified DL4 IBs were denatured with 8 M urea by incubating in boiling water for 5 min and immediately refolding was initiated by rapid dilution with 1X phosphate buffered saline (PBS) (pH 7.4). To assess the thermal stability of GFP variants, protein samples were incubated at different temperatures from 70°C to 90°C at 5°C intervals for 30 min and the remaining fluorescence was recorded. For time-dependent assay, protein samples were incubated at 80°C for 40 min with 10 min time intervals and the remaining fluorescence was recorded.

### Laser scanning confocal microscopic analysis

The *E. coli* expressing DL4 mutant and GFP-hs1 were grown at 37°C till mid-log phase and induced with 0.5 mM IPTG for 5 hours. 200 μl of the cells were harvested, centrifuged at 1200 g for 5 min and resuspended in 200 μl of 1X PBS. The cells were then fixed on glass slide with freshly prepared 4% paraformaldehyde (dissolved in PBS), then washed with 1X PBS and cover slip was placed for samples to be observed. The images were photographed at 488 nm using Plan-Apochromat objective (100X, NA 1.4 oil) in Zeiss LSM 510 confocal microscope (Carl Zeiss, Germany). The resulting image was analyzed using the color palette option in the Zeiss LSM image examiner.

### The thioflavin T assay, proteolytic digestion, and electron microscopy for IBs

Purified DL4 IBs were tested by thioflavin-T (ThT) binding assay in Tris-NaCl buffer (pH8.0). The reaction was carried out by incubating IBs in 25 μM of ThT and the emission was recorded after exciting at 450 nm. The amyloidogenic protein bound with ThT dye will give emission maxima at 480 nm [26,27]. Proteolytic digestion and electron microscope analysis of the purified IBs were carried out based on the reported methods [28,29]. The IBs diluted 20 times in Tris-NaCl buffer were treated with proteinase K (20 μg/ml) and digested overnight at 37°C. The solution was briefly centrifuged at maximum speed and the pellet was resuspended in distilled water. To observe under electron microscope, the sample was placed on carbon coated copper grid for 5 min at room temperature. The grid was rinsed with 10 μl of distilled water for 2 min and then the negative staining was done with 2% uranyl acetate for 1 min. After blotting and air drying, the electron micrograph images of inclusion bodies were captured using Hitachi transmission microscope (H-7600).

### Determination of particle size by light scattering

The size distribution of IBs was studied by dynamic light scattering using 90 Plus, Particle Size Analyzer, Brookhaven Instruments Corporation. Purified IBs suspended in 50 mM Tris (pH8.0) were sonicated for 1 min at room temperature. The samples were diluted 100 fold in the same buffer and the measurement was recorded at 25°C with dust filter turned on.

## Results

### Active IB formation of DL4 *in vivo*

The GFP mutant termed “DL4” was generated based on GFP-hs1, a GFP variant with high folding robustness and stability. The remarkably stable structure of GFP-hs1 was expected to be beneficial in tolerating for various mutations, which motivated us to construct various deletion mutations of GFP-hs1. It was possible to delete some internal loop sequences of GFP-hs1 without abolishing its folding and fluorescent activity, whereas those deletions led to the misfolding of normal GFP. Expression studies were carried out for the active deletion variants, which revealed that most of them were expressed as soluble forms. Unexpectedly, one variant (DL4) devoid of exposed loop sequence (191-GPVLLP-196) was identified to be expressed as insoluble forms with fluorescent activities. The primary structure of GFP-hs1 and DL4 were presented in supplementary material, Additional file 1: Figure S1. More details were described in our previous report [22].

**Figure 1:**
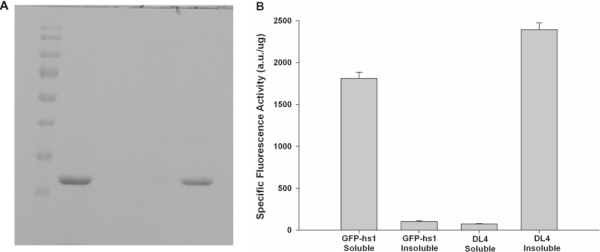
**Expression and functional analysis of GFP-hs1 and DL4. (A)** Soluble and insoluble expression profiles of GFP-hs1 and DL4 by SDS-PAGE. M – Marker, Lane 1,3 - soluble fractions of GFP-hs1 & DL4, Lane 2,4 – insoluble fractions of GFP-hs1 & DL4. **(B)** Specific fluorescence activities of DL4 and GFP-hs1 in their soluble and insoluble fractions. Specific fluorescence activity is defined as the fluorescence per microgram of protein. The values are the average of three measurements and standard deviations are shown.

**Figure 2:**
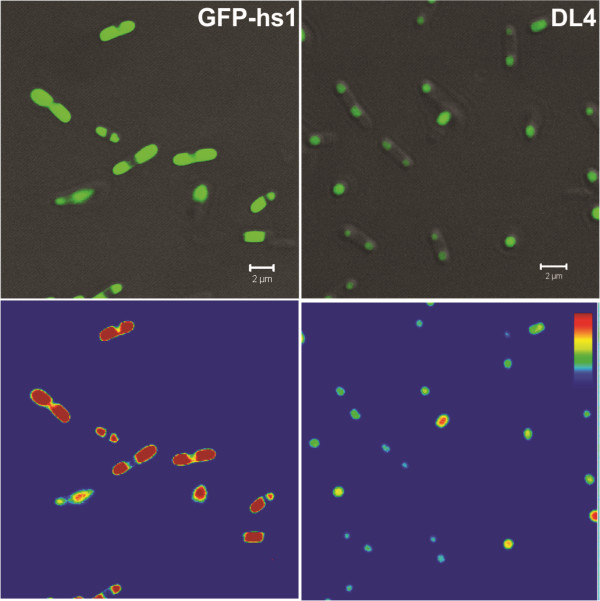
***In vivo*analysis of IBs formation.** (Top) Analysis of protein distributions in *E. coli* using confocal microscope. Protein aggregates were observed as dense molecules forming localized pattern of distribution (DL4) whereas the expression of soluble protein was spread uniformly over the cytoplasm (GFP-hs1). Scale bar represents 2 μm. (Bottom) The green fluorescence intensity mapped in color scale from low (blue) to high (red).

The unexpected observation that DL4 showed insoluble but fluorescent expression motivated us to check the possibility of the formation of active IBs. First, a study on the expression pattern of the DL4 was performed in detail. When GFP-hs1 and DL4 were expressed at 37°C in *E. coli* and their cell lysates were analyzed, DL4 protein was exclusively found in insoluble fraction with traces of DL4 in its soluble fraction, whereas GFP-hs1 was mostly found in the soluble fraction (Figure 1A). The specific fluorescence activities of the soluble and insoluble fractions of GFP-hs1 and DL4 also confirmed that the expressed DL4 was exclusively deposited into insoluble fraction in its highly active form, whereas active GFP-hs1 was expressed as soluble forms (Figure 1B). The specific fluorescent activities of GFP-hs1 and DL4 were almost negligible in their insoluble fractions and soluble fractions, respectively. The lower specific fluorescence activity of the GFP-hs1 in soluble fraction compared to the insoluble deposits of DL4 might be due to the presence of other cellular proteins in soluble fraction of GFP-hs1. To check whether the exclusive deposition of DL4 into insoluble fraction occurred right after *in vivo* protein translation or during the process of cell lysis, the intracellular states of DL4 and GFP-hs1 were investigated by *in vivo* image analysis with laser scanning confocal microscope. As shown in Figure 2, DL4 was observed as a dense fluorescent aggregates and it was confined to one corner of the bacterial cell. On the other hand, the control protein, GFP-hs1, was spread throughout the cytoplasm of the bacterial cell without any particulate morphology. The image analysis with fluorescence intensity color palette also showed the distinct difference in distribution of proteins between the GFP-hs1 and DL4. These results clearly indicated that the aggregation of DL4 occurred in bacterial cell after the protein translation and folding, confirming that the DL4 was exclusively expressed as intracellular active IBs in *E. coli*.

### Fluorescent properties of DL4 IBs

Next study was to examine the fluorescent properties of DL4 proteins in the IBs such as excitation/emission wavelength, quantum yield and specific fluorescent activity. These examinations were expected to provide following information. First was the effect of deletion mutations on the structural properties of GFP. The fluorescent properties of GFP are known to be very sensitive to the structural perturbation around active site of the protein, and can be used to probe the structural changes induced by mutations [13]. Second, the portion of active DL4 in the IBs, presumed to be highly dominant, would be more exactly estimated by comparing the spectral properties of DL4 IBs with GFP-hs1.

**Figure 3:**
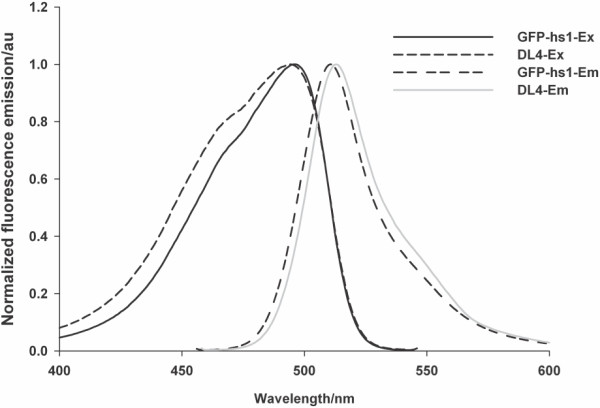
**Excitation/emission spectra of GFP-hs1 and DL4.** Protein samples were scanned for excitation (between 400 & 550 nm) and emission spectra (between 450 & 600 nm) using fluorescence spectrophotometer.

The excitation/emission wavelength and quantum yield, the representative intrinsic spectral properties of fluorescent proteins were estimated for the purified IBs of DL4, and they were compared with those of purified GFP-hs1. The spectral maxima for the excitation and emission were scanned for DL4 and GFP-hs1 (Figure 3), and both proteins showed almost similar fluorescent spectra with maxima ex/em at 490/511 nm (Table 1). The quantum yield of DL4 was determined to be about 0.33, also comparable value to GFP-hs1 (Table 1). These results indicated that the intrinsic spectral properties of GFP-hs1 were not affected by the deletion of 191–196 loop sequence. As mentioned above, the fluorescent properties such as ex/em wavelength and quantum yield are generally affected by the global GFP structure. Therefore, our results indirectly indicated that the DL4 structure may not be significantly different from its native form.

**Table 1:** Spectral and fluorescent properties of purified DL4 and GFP-hs1

**Protein** **sample**	**Excitation ** **peak (nm)**	**Emission peak (nm)**	Quantum yield*	Specific fluorescence Activity of purified proteins (a.u./μg)**
**GFP-hs1**	490	511	0.33 ± 0.02	5244.0 ± 237.69
**DL4**	490	511	0.33 ± 0.03	4978.5 ± 1062.78

*Average of three independent measurements represented with standard deviation.

**Average of two independent measurements represented with standard deviation.

The same fluorescent properties of DL4 and GFP-hs1 implied that the portion of the active DL4 proteins in the IBs can be estimated simply by comparing the specific fluorescence activity of purified DL4 IBs and that of purified GFP-hs1. The specific fluorescent activity of DL4 IBs was determined to be approximately 95% of GFP-hs1 (Table 1). Because the purified DL4 IBs were evaluated to include only DL4 variants by SDS-PAGE analysis (data not shown), this result indicated that the DL4 IBs was mostly composed of active DL4 proteins and the proportion of inactive DL4 proteins was negligible.

### Refolding kinetics and thermal stability of DL4

**Figure 4:**
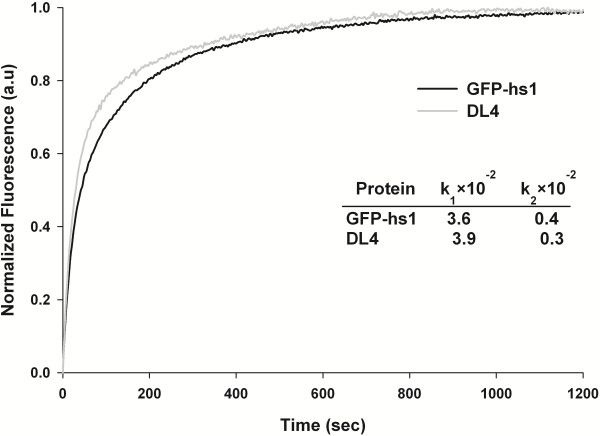
**Refolding kinetics of DL4 and GFP-hs1.** The plot shows the refolding progression curve of DL4 and GFP-hs1, measured after complete denaturation in urea followed by renaturation initiated rapidly by dilution. Inset table shows the refolding rates of GFP variants. Normalized fluorescence in arbitrary units (au) was plotted against time in seconds.

GFP-hs1, the template used for generating the DL4 variant, was constructed based on the previously reported mutations which were known to enhance the folding and stability of GFP [25,30,31]. The biophysical properties of GFP-hs1 have been well studied and the protein was evaluated to be highly stable as well as fold very efficiently under various protein denaturation conditions [32-34]. In above studies, DL4 and GFP-hs1 didn’t show any difference in their fluorescent properties at all, indicating that the deletion of loop introduced to DL4 didn’t affect the final global structure of GFP significantly. On the other hand, protein folding and stability of GFP were more sensitively affected by the introduced mutations according to our previous studies [35]. Here, the refolding kinetics and thermal stability of DL4 and GFP-hs1 were measured and compared in order to study the influence of the loop deletion in DL4 on the folding efficiency and stability of GFP-hs1. An *in vitro* refolding kinetics of each protein was performed using fluorescence spectrophotometer to compare the folding rates of DL4 and GFP-hs1. The protein samples were completely denatured by boiling in presence of a chaotropic agent urea and the refolding process was initiated by rapid dilution in PBS. The progression of protein refolding was examined by recording the increase in fluorescence emission of the GFP variants (Figure 4). The refolding rate of DL4 was found to be nearly similar to that of GFP-hs1.

**Figure 5:**
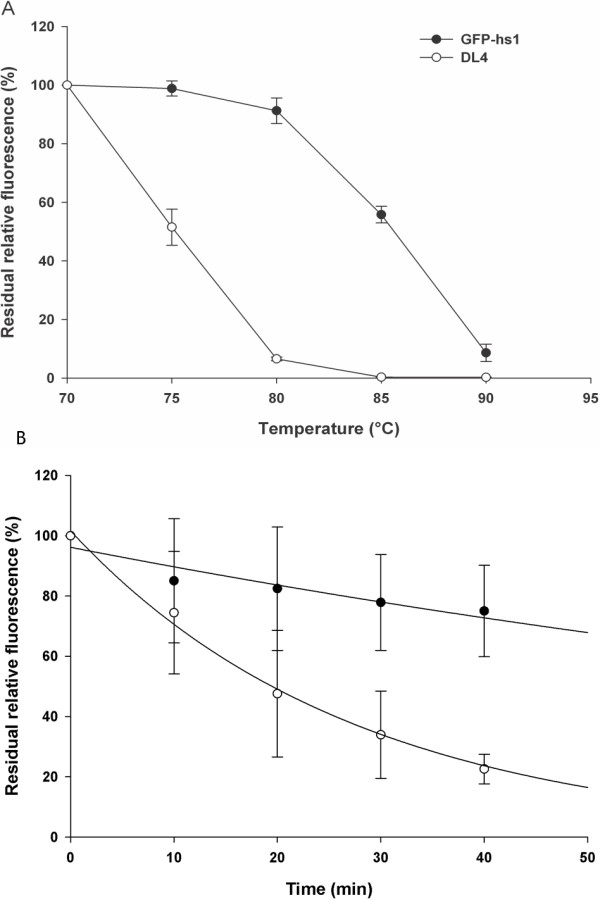
**Thermal stability of DL4 and GFP-hs1. (A)** Effect of various temperatures on the stability of DL4 and GFP-hs1. The stability was measured by incubating at different temperatures for 30 minutes and the remaining fluorescence was plotted against temperature as function. (Error bar – Standard deviation of three experiments). **(B)** Time-dependent temperature effect on the stability of DL4 and GFP-hs1 at 80°C. The fluorescence at time zero min was taken as 100%. (Error bar – Standard deviation of two experiments).

The stabilities of deletion mutant DL4 and control GFP-hs1 were investigated at different temperature. Specifically, the thermal stability was evaluated by incubating both protein samples at temperatures between 70°C and 90°C with 5°C increments for 30 min. The fluorescence remaining after heat treatment was measured using filter fluorometer and plotted as a function of temperature. As shown in Figure 5A, the activities of both DL4 and GFP-hs1 were not changed at 70°C for 30 min, but DL4 readily lost its fluorescence as the temperature increases compared to GFP-hs1 from 75°C. In time-dependent assay at 80°C, the fluorescence of DL4 was decreased much faster than that of GFP-hs1 as incubation time increases (Figure 5B). The inactivation constants of DL4 and GFP-hs1 were estimated to be approximately 0.036 min^-1^ and 0.007 min^-1^, when we evaluated them using the first-order inactivation model of GFP [36].

Overall, DL4 showed similar refolding kinetics, but lower stability compared to GFP-hs1. These results indicated that the deletion of loop sequence didn’t affect the folding of GFP-hs1, but destabilized the protein structure. Together with the similar spectral properties of DL4 and GFP-hs1, all of these observations support the hypothesis that DL4 may fold into its active native structure through almost similar pathway of GFP-hs1, but the native structure of DL4 might be unstable and prone to form the native-like self-aggregating species. The relation between the stability of DL4 and aggregation mechanism will be further discussed in Discussion section.

### Organization of DL4 in IBs

Misfolded protein mediated formation of IBs is generally known to form amorphous structures [37]. On the other hand, it was confirmed that some IBs had amyloid-like structures where inner molecules were relatively well-ordered by intermolecular β-strand interactions [27]. We examined whether the DL4 IBs composed of β-strand rich GFP molecules had well-organized inner structures by evaluating their amyloid-like structural properties. For this, two representative amyloid-structure specific assays were applied to the DL4 IBs.

**Figure 6:**
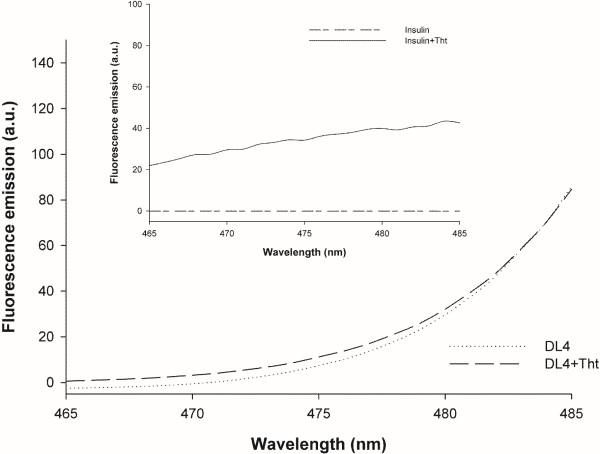
**Thioflavin T dye binding assay.** Emission spectra of DL4 aggregates with and without ThT binding. The excitation wavelength was 450 nm. Inset shows the emission spectra of insulin aggregates with and without ThT binding as positive control.

The thioflavin T (ThT) assay is the most commonly used method to check the presence of cross β-structure conformation in amyloid-like structures. The ThT selectively binds to amyloid protein and shows increase in fluorescence around 480 nm when excited at 450 nm [38]. DL4 IBs were treated with ThT dye and the emission spectrum was scanned after exciting at 450 nm. Insulin aggregates known to form amyloid-like structure was also tested as a positive control [39]. As shown in Figure 6, the intrinsic fluorescence of DL4 proteins caused the increase of fluorescence for the DL4 aggregates along the increase of emission wavelength, but the emission spectra of DL4 aggregates with ThT or without ThT exhibited no difference from 465 nm to 485 nm. The aggregated insulin proteins bound to ThT showed clear increase in fluorescence compared to the aggregates without ThT treatment. These results indicated the absence of ordered amyloid-like structure in DL4 aggregates.

**Figure 7:**
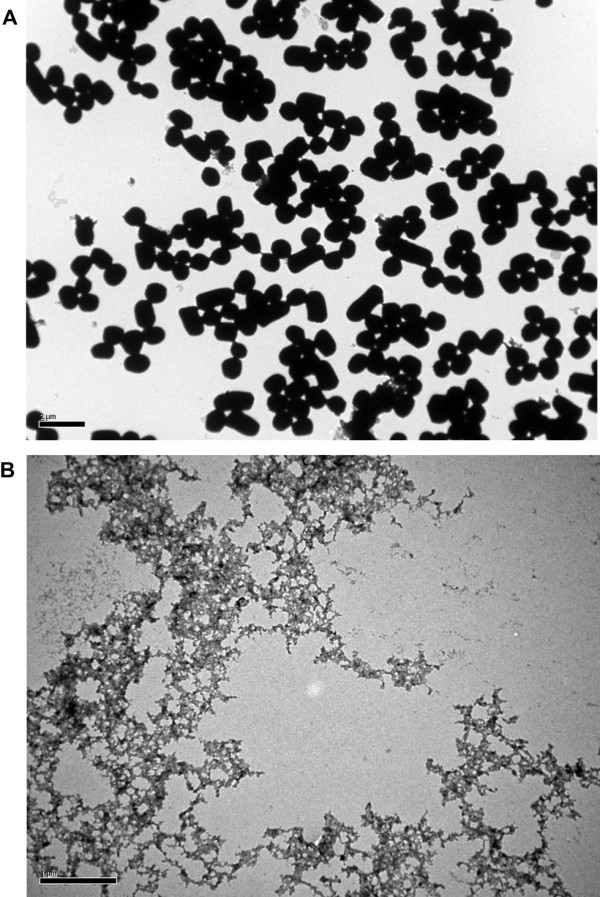
**Morphology of DL4 IBs before and after proteinase K treatment. (A)** Electron micrograph of purified DL4 IBs (scale bar 2 μm) **(B)** Electron micrograph of DL4 IBs after proteinase K treatment. The scale bar depicts 1 μm.

Proteolytic digestion of IBs containing amyloid-structure generally yields the formation of amyloid fibrils, another way to check the existence of amyloid-like structure [18]. To explore such structural morphology change of DL4 IBs, transmission electron microscopy (TEM) was also employed. The electron micrograph of purified DL4 IBs was found to display electron-dense, irregular sphere like shape (Figure 7A). The IBs was treated with proteinase K, which did not reveal any structural morphology pertaining to amyloid fibril (Figure 7B). This also supported that the DL4 IBs might not be an ordered amyloid-like conformation, but disordered amorphous structure.

### Preparation of IB-based fluorescent nanoparticles

**Figure 8:**
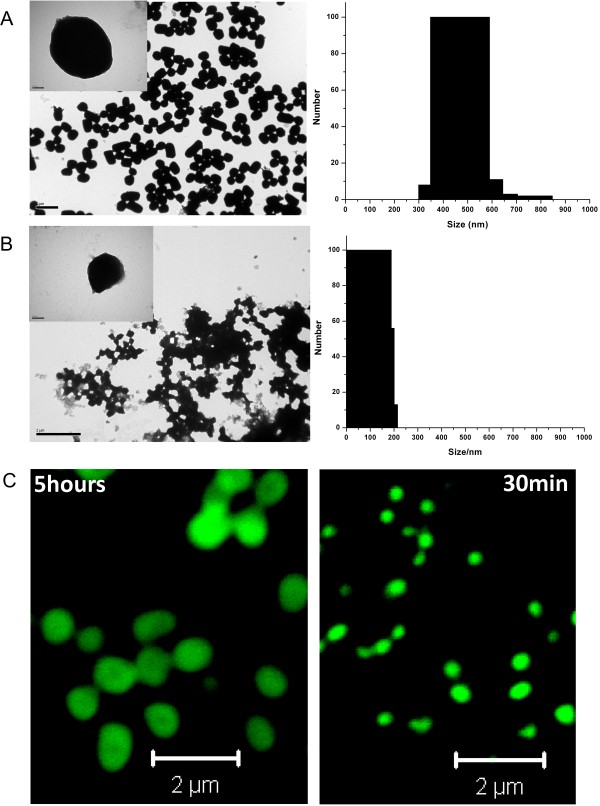
**Fluorescent protein nanoparticles based on DL4 IBs.** Particle sizes and fluorescent images of purified IBs were analyzed by electron microscope, dynamic light scattering and confocal imaging. **(A)** Negative stained TEM image and DLS data of the purified DL4 expressed for 5 hours **(B)** Negative stained image and DLS data of purified DL4 expressed for 30 min. (Scale bar 2 μm). Inset figures show single IB of DL4 (scale bar 100 nm). **(C)** Confocal microscope images of the purified fluorescent DL4 particles expressed for 5 hours and 30 min (Scale bar 2 μm).

Fluorescent protein nanoparticles can be used in bio-imaging, *in vivo* tracking of biomolecules and as tracers for intravascular imaging [40,41]. Here, an attempt was made to reduce the particle size of fluorescent DL4 IBs to explore the possibility of utilizing the biologically active DL4 IBs as fluorescent nanoparticles. This was attempted by regulating the expression time of the recombinant protein at 37°C in *E. coli*. DL4 was expressed for 5 hours and 30 min, and the particle size distribution and structural geometry of purified proteins were analyzed by dynamic light scattering (DLS) and transmission electron microscope (TEM). As shown in Figure 8A, the size of harvested IBs after 5 hours of expression were in the range of 500 nm to micrometer, which was the most common morphological size of many bacterial inclusion bodies [11,42]. On the other hand, the IBs from the cells expressing the DL4 for 30 min produced the particles around 100–200 nm in diameter (Figure 8B). Specifically, the particle size distribution of IBs by DLS analysis showed the reduction in size diameter from 400–600 nm to < ~200 nm when the expression time was lowered from 5 hours to 30 min, as shown in bar diagram of Figure 8A & B. The TEM results were roughly correlated with results of DLS measurements, indicating the major population of IBs produced at 5 hours and 30 min were around 500 nm and 100–200 nm in diameter respectively. The functional analysis of the DL4 IBs from 5 hours and 30 min expression by confocal microscopy also showed green fluorescence emission and exhibited distinct differences in their particle size diameter (Figure 8C). These results suggested that the size of bio-functional fluorescent DL4 IBs could be reduced through simple modulation of expression times, which might enable the preparation of fluorescent protein nanoparticles.

## Discussion

Active GFP IBs, potential fluorescent biomaterials, can be simply achieved by over-expressing a GFP with low folding efficiency in bacteria [14]. The GFP polypeptide chains are generally misfolded, aggregated and deposited to IBs. Such IBs exhibit fluorescence because correctly folded active GFP molecules are aggregated together with the misfolded proteins, but the major proteins in the IBs are misfolded non-fluorescent proteins. The formation of bacterial IBs including more native fluorescent GFPs can be induced by fusing self-aggregating/assembling peptide tags to GFP terminus [16,19]. In this study, we have proposed another version of active GFP IBs induced by the DL4, a GFP variant devoid of some loop residues. DL4 formed exclusively active IBs in *E. coli* through its intrinsic self-aggregating property, and the IBs was confirmed to be composed of mostly active fluorescent proteins. To our knowledge, DL4 is the first GFP variant which forms exclusively active intracellular IBs without any selective peptide or protein tags for the self-aggregation/assembly.

Our study has indicated that the DL4 protein folds to be its active form and the folded structures are aggregated inside the cell. What induces the self-aggregation of the folded DL4 proteins? In our previous study, the dynamic nature of some exposed residues in DL4 structure was confirmed through molecular dynamics simulation [22]. Based on the results, we proposed that the introduced deletion mutation in DL4 marginally destabilized the folded protein structure, which might induce the self-aggregation of the folded protein. In the current study, DL4 showed lower thermal stability compared to its native GFP, which demonstrates the destabilization effect of deletion mutation. This supports the hypothesis of destabilization effect on the self-aggregation of DL4 indirectly. Further detailed studies should be performed to understand the self-aggregation mechanism of DL4 more exactly.

An impression from our study is that biologically active IBs can be achieved through engineering a protein sequence itself. As mentioned in the introduction section, active IBs have great potentials as biocatalysts and biomaterials. A major concern is how to generate such active IBs for target proteins. Most studies on the active IBs generally relied on the protein’s natural property of active aggregation or tagging some aggregating peptide/protein sequences to the protein sequence [6,16]. Engineering of protein sequence itself has not been seriously considered to generate a mutant for active IBs presumably because there might be a preconception that protein sequence engineering which can induce the protein aggregation may be possible only by inducing non-functional misfolded proteins. On the other hand, the DL4 was generated by deletion of GFP sequence, which suggests that we can generate an active aggregating mutant through engineering of a protein native sequence. Although it is quite hard to generalize such sequence engineering approach for the generation of active IBs, partial and marginal destabilization of a target protein can be a way to generate such mutants as discussed above.

In this study, it was demonstrated that fluorescent protein particles could be prepared by simply expressing the DL4 in *E. coli*. The size of fluorescent IB particles could be reduced up to around 100–200 nm by decreasing the DL4 expression time. However, the DL4-based protein particle size needs to be further reduced to use them more efficiently for real applications such as fluorescent nanotracers. In fact, we tested the possibility whether the particle size could be further reduced by lowering the expression time or lowering the expression temperature further. The problem in this attempt was that the produced fluorescent IBs were too sticky to be handled, which hampered the further characterization. The overcoming of this limitation may facilitate the use of DL4-based particles.

## Conclusions

In this study, we have studied various biophysical properties of a GFP variant (DL4), and its IBs. It was demonstrated that the DL4 was exclusively produced as active protein aggregates under *in vivo* condition through its spontaneous self-aggregation property. The intrinsic property of DL4 to form aggregates *in vivo* was exploited to produce fluorescent protein particles with different sizes by modulating the expression time. It is expected that DL4 IBs can be used as fluorescent biomaterials. We also expect that DL4 can be employed as a potential model protein to understand the aggregation mechanism of native-like proteins present in active IBs, which might give probable clues on the protein aggregation related diseases.

## Competing interests

The authors declare that they have no competing interests.

## Authors’ contributions

GR and SL designed the research. GR, GM and HM performed the experiments. GR, GM and SL analyzed the data and wrote the paper. HP, SSAA, YK and SK provided the experimental and analytical tools and revised the manuscript. All authors approved the final manuscript.

## Supplementary Material

Additional file 1: Figure S1 Amino acid sequences of GFP-hs1 and DL4 with His-tag at C-terminal end. Deletion of loop region of DL4 involves six residues (GPVLLP) and they are highlighted in bold with yellow background in primary sequence of GFP-hs1. An additional amino acid glutamine “Q” has been placed after first amino acid methionine in both constructs. Click here for file
