# Supplementary material for: A variant of green fluorescent protein exclusively deposited to active intracellular inclusion bodies
Source: Microb Cell Fact. 2014 May 16;13:68. doi: 10.1186/1475-2859-13-68 (PMC4049505; doi:10.1186/1475-2859-13-68)
Supplement: Additional file 1: Figure S1 — Amino acid sequences of GFP-hs1 and DL4 with His-tag at C-terminal end. Deletion of loop region of DL4 involves six residues (GPVLLP) and they are highlighted in bold with yellow background in primary sequence of GFP-hs1. An additional amino acid glutamine “Q” has been placed after first amino acid methionine in both constructs. [file 1475-2859-13-68-S1.docx]

>GFP-hs1

MQSKGEELFTGVVPILVELDGDVNGHKFSVRGEGEGDATNGKLTLKFICTTGKLPVPWPT

LVTTLGYGVQCFARYPDHMKRHDFFKSAMPEGYVQERTISFKDDGTYKTRAEVKFEGDTL

VNRIELKGIDFKEDGNILGHKLEYNFNSHKVYITADKQKNGIKANFKIRHNVEDGSVQLA

DHYQQNTPIGD**GPVLLP**DNHYLSTQSVLLKDPNEKRDHMVLLEFVTAAGITHGMDELYKH

HHHHH

>DL4

MQSKGEELFTGVVPILVELDGDVNGHKFSVRGEGEGDATNGKLTLKFICTTGKLPVPWP

TLVTTLGYGVQCFARYPDHMKRHDFFKSAMPEGYVQERTISFKDDGTYKTRAEVKFEGDT

LVNRIELKGIDFKEDGNILGHKLEYNFNSHKVYITADKQKNGIKANFKIRHNVEDGSVQL

ADHYQQNTPIGDDNHYLSTQSVLLKDPNEKRDHMVLLEFVTAAGITHGMDELYKHHHHHH

**Figure S1:** Amino acid sequences of GFP-hs1 and DL4 with His-tag at C-terminal end. Deletion of loop region of DL4 involves six residues (GPVLLP) and they are highlighted in bold with yellow background in primary sequence of GFP-hs1. An additional amino acid glutamine “Q” has been placed after first amino acid methionine in both constructs.
